# Integrating Demographics and Imaging Features for Various Stages of Dementia Classification: Feed Forward Neural Network Multi-Class Approach

**DOI:** 10.3390/biomedicines12040896

**Published:** 2024-04-18

**Authors:** Eva Y. W. Cheung, Ricky W. K. Wu, Ellie S. M. Chu, Henry K. F. Mak

**Affiliations:** 1 School of Medical and Health Sciences, Tung Wah College, 31 Wylie Road, HoManTin, Hong Kong; 2 Department of Biological and Biomedical Sciences, School of Health and Life Sciences, Glasgow Caledonian University, Glasgow G4 0BA, UK; 3 Department of Diagnostic Radiology, School of Clinical Medicine, LKS Faculty of Medicine, University of Hong Kong, Hong Kong

**Keywords:** Alzheimer’s disease, mild cognitive impairment, dementia, radiomics, volumetry, feed forward neural network, artificial neural network

## Abstract

Background: MRI magnetization-prepared rapid acquisition (MPRAGE) is an easily available imaging modality for dementia diagnosis. Previous studies suggested that volumetric analysis plays a crucial role in various stages of dementia classification. In this study, volumetry, radiomics and demographics were integrated as inputs to develop an artificial intelligence model for various stages, including Alzheimer’s disease (AD), mild cognitive decline (MCI) and cognitive normal (CN) dementia classifications. Method: The Alzheimer’s Disease Neuroimaging Initiative (ADNI) dataset was separated into training and testing groups, and the Open Access Series of Imaging Studies (OASIS) dataset was used as the second testing group. The MRI MPRAGE image was reoriented via statistical parametric mapping (SPM12). Freesurfer was employed for brain segmentation, and 45 regional brain volumes were retrieved. The 3D Slicer software was employed for 107 radiomics feature extractions from within the whole brain. Data on patient demographics were collected from the datasets. The feed-forward neural network (FFNN) and the other most common artificial intelligence algorithms, including support vector machine (SVM), ensemble classifier (EC) and decision tree (DT), were used to build the models using various features. Results: The integration of brain regional volumes, radiomics and patient demographics attained the highest overall accuracy at 76.57% and 73.14% in ADNI and OASIS testing, respectively. The subclass accuracies in MCI, AD and CN were 78.29%, 89.71% and 85.14%, respectively, in ADNI testing, as well as 74.86%, 88% and 83.43% in OASIS testing. Balanced sensitivity and specificity were obtained for all subclass classifications in MCI, AD and CN. Conclusion: The FFNN yielded good overall accuracy for MCI, AD and CN categorization, with balanced subclass accuracy, sensitivity and specificity. The proposed FFNN model is simple, and it may support the triage of patients for further confirmation of the diagnosis.

## 1. Introduction

With an increasingly aging global population, the incidence of dementia is rapidly increasing. In 2016, there were 47 million people living with dementia worldwide. This figure is projected to increase to more than 131 million by 2050 [1]. The most common cause of dementia is Alzheimer’s disease (AD), which accounts for approximately 40% of all dementia cases. With recent pharmacological advancements, drug therapies for ameliorating the progression of AD [2] and improved preventive measures and therapies for AD have been developed. The early detection and accurate diagnosis of the prodromal stage of dementia, i.e., mild cognitive impairment (MCI), are crucial to reduce mortality, improve the quality of life and extend the lifespan of patients with dementia.

MRI magnetization-prepared rapid gradient-echo (MPRAGE) imaging is a down-stream imaging modality, which captures high tissue contrast with superior spatial resolution in a short scan time [3]. The three-dimensional application of whole-brain scans has been extensively used for AD diagnosis and disease progression monitoring. It provides detailed structural images of the brain, allowing physicians to visualize and assess the brain abnormalities associated with dementia. It is easily available and plays a crucial role in dementia diagnosis. 

One of the MRI MPRAGE image applications is brain volumetric analysis. A significant volume reduction in the medial temporal lobe, including the hippocampus, precuneus, posterior cingulate, amygdala, parahippocampal gyrus and entorhinal cortex, is a signature for AD patients [4,5,6,7,8]. Through detailed hippocampal volume assessment [9,10], sub-regional corpus callosum atrophy [11,12] and connectivity-based segmentation of amygdala nuclei [13], AD can be effectively diagnosed from a cognitive normal (CN) state. Recent developments in automatic brain regional volume segmentation have improved the segmentation accuracy and can handle large amounts of data effectively. This allows for the comprehensive analysis of yearly MRI MPRAGE images for disease monitoring. Previous studies suggested that AD progression can be predicted based on the rate of volume reduction by monitoring the hippocampal volume change [14,15]. However, for the prodromal stage of AD, which is MCI, the brain regional volume change is subtle and cannot be easily detected by the naked eye. The diagnosis of MCI from AD requires either supplements with an up-stream imaging modality or extensive experience and knowledge from clinical experts. Neither of them is commonly available in memory clinics.

In recent years, the radiomics analysis of MRI MPRAGE has been widely applied in medical imaging. It is a novel technique that incorporates gray-level invariant features (GLIFs) into a data classification algorithm. It has the potential to reveal disease heterogeneity characteristics, which are related to the gray-level matrixes. This method has been adopted for cancer prognosis and recurrence prediction [16,17,18], the prediction of distant metastasis [19] and treatment response [20]. In view of dementia classification, an exploratory study was conducted by Li et al. 2020 using pure radiomics, and 55.9–56% accuracy was achieved in diagnosing preclinical AD. However, the accuracy improved to 76.1% when combined with other high-frequency features [21].

Biological differences and aging are other perspectives on dementia development. Previous studies suggested that women in many cohorts have a higher risk of developing AD [22,23]. Also, a higher incidence of dementia in elderly individuals is observed around the world, and the prevalence ranges from 5 to 7%, even after age standardization [24]. Including age and sex as parameters in the prediction model may have a positive impact on discriminating AD, MCI and CN in different perspectives.

During the past two decades, many studies have applied artificial intelligence to dementia classification using traditional classifiers, including logistic regression [25,26], decision tree (DT) [27], random forest [28,29,30], naïve Bayes [31], K-nearest neighbor [32], support vector machines (SVMs) [33,34,35,36,37,38] and ensemble classifier (EC) [39]. With the improvements in computer processing power, more studies have focused on using discriminative approaches, such as neural networks, in recent years. It is a branch that simulates the human brain, both in terms of structural and learning patterns. Compared to the traditional classifier, it allows for the processing of complicated high-level information by connecting a large number of inputs [40]. In addition, a multiple-layer neural network can capture complex non-linear relationships in data, as well as learning the relevant features automatically. The feed-forward neural network (FFNN) is one of the most popular neural networks being employed. It processes information from the input layer, through hidden layers to the output layer in one direction, without any feedback connections. It has only a few hidden layers, which requires less computation power to process, and is able to provide a good classification with a smaller dataset when compared to deep learning models. Previous studies showed good accuracies in identifying AD from CN (>85%) and MCI from CN (>80%) [41,42,43,44]. However, most studies relied on a single dataset to train and test the model. The models were not tested against unseen data, which may affect the generalizability of the built model and limit its application in clinical settings. Also, a binary classifier, i.e., to classify AD from CN or MCI from CN, was employed in most studies. In real-world scenarios, patients’ images were retrieved from multiple stages. The classification may be required to fit patients’ images into several binary classifiers to confirm the diagnosis. Instead of training and managing the multiple binary classifiers of each class, a multi-class classifier is designed to handle multiple classes simultaneously. Although it is more challenging to train and yields lower accuracy [45], the deployment of the multi-class model provides only one output for various stages of diseases. It is simple and efficient to precisely identify these diseases. 

In this study, we aimed to develop a reliable artificial intelligence multi-class model to classify AD, MCI and CN using patient demographics and MRI imaging features. The first objective was to use various combinations of demographics and image features to build the models using FFNN and various traditional artificial intelligence algorithms, including DT, EC and SVM, as a comparison. The second objective was to compare the classification performances of the FFNN with those of the above-developed models to identify the algorithm that could provide a more accurate classification of AD, MCI and CN.

## 2. Materials and Methods

In this study, two cohorts of patients were used to build and validate the artificial intelligence models. For each patient, the demographics were recorded. In addition, the brain regional volumes, as well as radiomics from the whole brain, were retrieved from the MRI images as image features. The patients’ demographics, brain regional volumes and radiomics were integrated as inputs for model building using FFNN, DT, EC and SVM algorithms. The model classification performances were analyzed. 

### 2.1. Patient Dataset

The datasets used in this study are the Alzheimer’s Disease Neuroimaging Initiative (ADNI) database (adni.loni.usc.edu) [46] and the Open Access Series of Imaging Studies (OASIS) database (oasis-brains.org) [47]. The use of the above datasets was approved by the institutional review board at each site, and all participants provided written consent. All eligible participants underwent brain MRI MPRAGE scanning and clinical diagnosis with demographics collected. 

#### 2.1.1. ADNI Dataset

There were 25 memory centers from the USA which joined the ADNI project. A total of 582 images were collected from 25 centers. Further, 406 images (70% of all images) from 21 memory centers were partitioned as the training dataset, and 176 images (30% of all images) from the remaining 4 centers were used as validation datasets. The distribution of images is listed in Table 1. 

#### 2.1.2. OASIS Dataset

An independent cohort dataset (OASIS dataset) was collected from the Washington University Knight Alzheimer Disease Research Center. The entire OASIS dataset consists of 1552 patients. Thus, 176 patients, 28 AD, 91 MCI and 57 CN, were picked randomly. The total number of patients and the distribution of subclasses were the same as the testing dataset from ADNI 4 centers. This was to ensure the result of testing using dataset from ADNI 4 centers and that using the OASIS dataset would not be influenced by the number of patients and its subclass distribution. 

### 2.2. Brain Segmentation and Regional Volume Analysis

**Figure 1:**
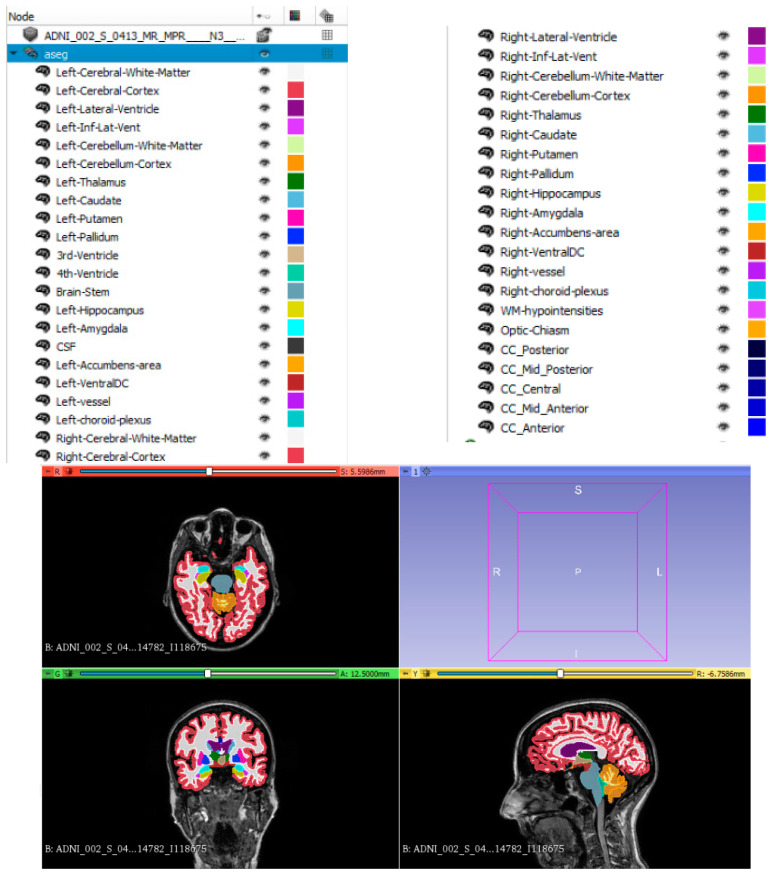
Extraction of 45 brain regional volumes in the software. Inf: inferior; Lat: lateral; Mid: middle; DC: diencephalon; WM: white matter; CC: corpus callosum.

FreeSurfer v7.1.0 image analysis suite was employed to perform brain segmentation and volumetric analysis. The procedures and algorithms employed were documented in previous publications [48,49,50,51,52,53,54] and are freely available from the website (http://surfer.nmr.mgh.harvard.edu/ (accessed on 22 January 2023)). Forty-five brain regional volumes were obtained. Details of the brain regions are listed in Table 2 and illustrated in Figure 1. 

### 2.3. Radiomics Features

**Figure 2:**
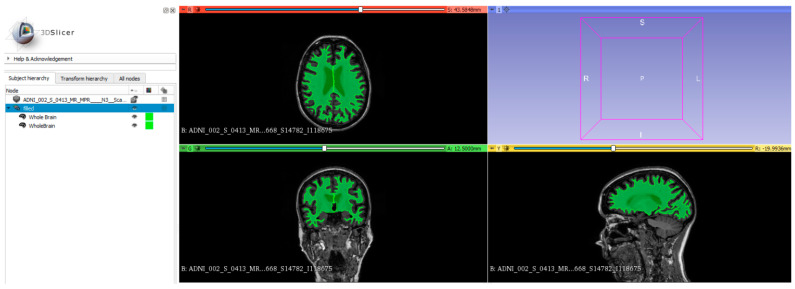
Individualized whole-brain mask (the green region) was used to quantify the whole brain for the retrieval of 107 radiomics features.

Reorientation of images was performed for each of the MPRAGE MRI images by SPM12 software [55]. The individualized whole-brain mask template was fused onto the MPRAGE image for brain regional configuration, which is shown in Figure 2. Further, 3D slicer software (The Slicer Community; V.4.11.20210226) with the PyRadiomics extension (Computational Imaging and Bioinformatics Lab, Harvard Medical School) was employed for the radiomics feature extraction [56]. One hundred and seven radiomics features were extracted within the whole brain from the MRI MPRAGE image for every patient. The definition of radiomics features was subdivided into eight classes [57], which are listed in Table 3. 

### 2.4. Demographics

The MRI MPRAGE dataset and patients’ demographics were retrieved from the ADNI and OASIS website. The demographics of age and sex were recorded.

### 2.5. Integration of Patients’ Demographics and Image Features

The patients’ demographics, brain regional volumes and radiomics were integrated in the following 5 groups, which were used as inputs for building the artificial intelligence models: radiomics only with 107 features (R only), radiomics and patents’ demographics with 109 features (RD), volumes only with 45 features (V only), volumes and patients’ demographics with 47 features (VD) and volumes, radiomics and patients’ demographics with 154 features (VRD). Details are listed in Table 4.

### 2.6. Model Building 

Patients from the ADNI dataset of 21 centers were used to build the models. The 5 groups of features obtained in Section 2.5 were used as input to build the models.

**Figure 3:**
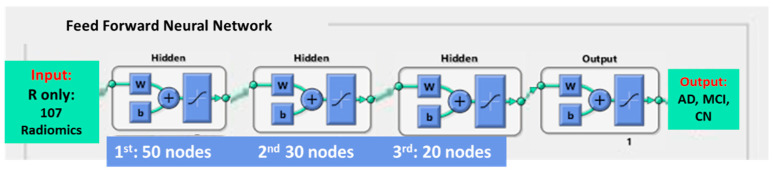
Details of the feed-forward neural network.

The proposed FFNN was built using Matlab^®^ (R2021a) Neural Network toolbox. The neural network training employed Levenberg–Marquardt as a training algorithm with the random data division method. It had 5 layers, including 1 input layer, 3 hidden layers and 1 output layer. The input layers were the 5 groups of features in Section 2.5. The 3 hidden layers included 50 nodes in the first layer, 30 nodes in the second layer and 20 nodes in the last hidden layer for processing. In each hidden layer, the weight (w) and bias (b) are valued as a single vector, as shown in Figure 3. The FFNN is trained to fit input data; then, its weight and bias values are formed (+) into a vector (curve in the diagram) and fitted to the next layer. The output layer gave the result of classification. In this model, three subclasses, either AD, MCI or CN, were classified.

During model building, the hyper-parameter optimization algorithm was employed to control the learning process so as to optimally solve the problem. The maximum epoch was set to 50, and no training time limit was applied. The three-layered FFNN was trained using mean squared error performance function and a regularization value of 0.01. This was the early stopping-based optimization, which was used to stop training when the performance function and a regularization value of 0.01 were achieved. Details of the FFNN model building are listed in Figure 3.

In addition, the Matlab Classification Learner toolbox was employed to build the models using traditional artificial intelligence algorithms, including DT, EC and SVM, as a comparison. Hyper-parameter tuning was employed, with Bayesian optimization as optimizer, expected improvement per second plus as the acquisition function, the maximum iterations set as 30 and no training time limit applied, in DT, EC and SVM model building, so as to reduce the instability and provide simple models [58].

To improve the generalizability of the built models and avoid overfitting, 10-fold cross-validation was employed during each of the model-building processes. The dataset was divided into ten groups with an equal number of samples. The first neural network training process used the initial nine groups as training data and the remaining group as testing data. The second training process continued with another nine groups as training data and the rest as testing data. This process was undertaken 10 times. The performance of each model was the average result computed in these 10 rounds of training [59]. 

The performance of each model was assessed in terms of the overall accuracy, the classification ability of each subclass, i.e., MCI, AD and CN by class accuracy, sensitivity and specificity.

### 2.7. Model Testing and Data Analysis

Each model was tested using two independent cohorts of patients, including patients from the 4 centers of the ADNI dataset and those from the OASIS dataset. The performance of each model was assessed considering the overall accuracy, the classification ability of each subclass, i.e., MCI, AD and CN by class accuracy, sensitivity and specificity. 

## 3. Results

We used five groups of features (R only, V only, RD, VD and VRD) to build models using four algorithms (FFNN, DT, EC and SVM); as a result, 20 models were built. Firstly, the value of the integration of multiple features was assessed through a performance evaluation using the same algorithm, with various features included in building the models. Secondly, the performance of the proposed FFNN was evaluated for various stages of dementia classification. 

### 3.1. Dataset Demographics

The ADNI dataset comprises patients from 25 centers. Further, 406 patients from 21 centers (ADNI 21 centers) were selected to build the model, and 176 patients from the remaining 5 centers (ADNI 5 centers) were used to test the model. Another independent dataset from the OASIS database was used, with 176 patients used for secondary validation on the models built in Section 2.6. The demographics of the study cohort are shown in Table 5.

### 3.2. Performance Comparison in View of the Various Features Employed for Model Building

When comparing models built using the same model-building algorithm, those models built using volumes performed better, with higher overall accuracy, accuracy in characterizing MCI, AD and CN, sensitivity and specificity when compared to those models built using radiomics. Including demographics as features for either volumes or radiomics improved the overall accuracy when compared to the use of volume or radiomics alone. However, in SVM algorithms, the specificity of AD classification was zero when using VD or VRD features. Overall, in all models, the integration of volumes, radiomics and demographics attained the highest overall accuracy, balanced sensitivity and specificity, as well as the best accuracy in classification in MCI, AD and CN. The results are listed in Table 6.

### 3.3. Performance Evaluation of FFNN when Compared to Traditional Classifiers 

The results from Section 3.2 suggest that the models using features from volumes, radiomics and demographics achieved the highest overall accuracy when compared to those built from either volumes or radiomics alone. Thus, we focused on analyzing models using all three features. In Table 7 e, it can be seen that the performance of FFNN was the best when compared to traditional classifiers. FFNN showed 76.57% and 73.14% overall accuracy in tests for patients from ADNI 4 centers and the OASIS database, respectively. In particular, the FFNN model attained good sensitivity and specificity.

## 4. Discussion

### 4.1. The Value of Integrating Image Features and Patient Demographics in AD, MCI and CN Classification

Our previous study suggested that structural MRI images aided in differentiating AD and MCI from CN using artificial intelligence [30]. However, that study was limited to binary classification, i.e., differentiating AD from CN, AD from MCI or MCI from CN. In clinical situations, CN may progress to MCI and then to AD in a matter of years. Multi-class classification is more useful considering three stages of disease. For two decades, brain regional volumes have been employed to diagnose AD from CN. Hippocampal atrophy is a widely used biomarker for the diagnosis of AD, but the low sensitivity and specificity limit its application as a confirmation of diagnosis [60]. Sørensen and his team suggested using other imaging features, including cortical thickness, hippocampal shape and its texture for the differential diagnosis of MCI from AD, and they achieved a classification accuracy of 62.7% for CN from AD and MCI [61]. Similar results were obtained by Koikkanlainen and his team, where 74% of AD could be accurately classified from other types of dementia using structural MRI [62]. Both authors suggested that other features might be required to attain higher accuracy in classification. Our results demonstrated that, using the volumes of only 45 brain regions, the overall classification accuracy achieved was 73.14% and 68% (EC) in validation for patients from ADNI 4 centers and OASIS, respectively. The results are similar to those obtained in previous studies. In subclass classification, however, the sensitivity was under 70% in AD and CN for all four algorithms. This suggested that the models built using brain regional volumes alone were unsatisfactory in identifying AD from CN. 

In recent years, radiomics has been employed in the classification of AD, MCI and CN. Du and his team used radiomics features of the hippocampus for diagnosing early-onset and late-onset AD, which achieved 77% and 78%, respectively. However, their sample size was small, with only 144 patients included in training (36 patients in each group) and another 60 patients (15 patients in each group) for testing [63]. The limited sample size may restrict the generalizability of the classification model. Our results demonstrated that, using radiomics as the only feature for model building, the overall accuracy achieved 40.57% (SVM) to 51.43% (FFNN) in tests using patients from ADNI 4 centers and 35.43% (SVM) to 58.29 (EC) for patients from OASIS, respectively. The models built using radiomics alone were well below satisfactory. 

To improve the classification accuracy, previous studies suggested building the model using multiple image features. Li and his team included 30,128 image features, including 24,910 features from structural MRI, 4988 features from functional MRI and 200 features from MRI Diffusion Tensor Imaging (DTI). They achieved overall accuracy of 90.2% and sensitivity and specificity of 79.8% and 86%, respectively [21]. The current study included only structural MRI image features, and, by adding patients’ demographics to the brain regional volumes and radiomics, the overall accuracy improved to 76.57% and 73.14% (FFNN) in tests for patients from ADNI 4 centers and OASIS, respectively. Also, the accuracy of MCI, AD and CN was 78.29%, 89.71% and 85.14% in tests for patients from ADNI 4 centers, which remained consistently high in tests for OASIS patients (74.86%, 88% and 83.43%). 

In addition to the overall accuracy, the sensitivity and specificity, which refer to a model’s ability to classify patients with AD as AD, and to classify patients who were not AD as MCI and CN, respectively, were balanced. This illustrated that the model demonstrated high capability in classifying the corresponding groups accurately. To address the issue of an imbalanced subclass dataset, we used the precision and F1 score to evaluate the models. Precision was used to measure how many predictions for one group made by the model were correct. Recall was used to measure the number of one-class samples present in the dataset that were correctly identified by the model. The F1 score combines precision and recall using their harmonic mean. The high F1 score illustrates maximized precision and recall simultaneously. In the current study, FFNN demonstrated the highest precision and F1 score when compared to other algorithms, which demonstrated that the FFNN model can concurrently attain high precision and high recall, indicating well-balanced performance.

### 4.2. The Value of the Feed-Forward Neural Network in Classification of AD, MCI and CN

In this study, the FFNN showed the best performance in terms of accuracy, specificity and sensitivity for dementia classification when compared to other traditional algorithms. FFNN is a multi-layer artificial neural network, with a connection between the input layer, hidden layers and output layers. The training process allows information to move in one direction, from the input layer and hidden layers to the output layer, without looping (backpropagation) [64]. This simulates the thinking process of a physician in clinical decision making and diagnosis confirmation, based on information from patients’ demographics and imaging features. 

The FFNN networks built in this study were relatively small in view of network training. There were only five layers (one input layer, three hidden layers and one output layer). The processing time is within 2 min when running on most computers in the clinical setting. The Levenberg–Marquardt algorithm used in FFNN offered significant accuracy, with fewer errors during the training, validation and testing phases [65].

### 4.3. The Value of Multi-Classes in Classification of AD, MCI and CN

Previous studies achieved good classification accuracies; for example, Zhang et al. 2019 achieved 96% accuracy in discriminating AD from CN, with sensitivity and specificity of 89% and 98%, respectively [66]. In addition, Mendoza-Leon and his team developed an auto-encoder model, which achieved accuracy of 90%, with sensitivity and specificity of 85% and 95%, respectively, in discriminating AD from CN [67]. Ning and his team demonstrated over 95% accuracy in classifying AD from CN [43]. A previous study from our team also achieved excellent classification accuracy, with 99.92% in differentiating MCI from CN, 99.86% to differentiate MCI from AD and 99.94% to differentiate AD from CN. However, these models were binary classifiers [30]. In real-world scenarios, however, patients can be taken from either stage. The multi-class model is a one-stop model, which can differentiate AD, MCI and CN distinctively. Technically, Borchert et al. 2023 highlighted that building a multi-class classifier model is more challenging than a binary classifier in view of the machine learning algorithm, and it usually yielded lower accuracy, sensitivity and specificity [68]. Compared to similar studies—one conducted by Moore and his team, where their model achieved accuracy of 99%, 59% and 29% in CN, MCI and AD, respectively [69], and another study conducted by Cárdenas-Pẽna and his team, where their model achieved 71.4%, 53.4% and 75.1% in CN, MCI and AD, respectively [42]—our proposed FFNN with VRD features yielded 83.43% 74.86% and 88% accuracies in CN, MCI and AD, respectively, in the OASIS test dataset. The balanced accuracies in various stages demonstrated that the model has the capability to classify all three stages of disease with satisfactory results, leading to a precise stage classification in real-world scenarios. 

### 4.4. The Value of Testing against Independent Cohort of Patients

Another important asset of the current study is the use of an independent dataset for validation. Compared to those studies using cross-validation or other similar methods, the use of an independent dataset demonstrated much lower accuracy [70]. For instance, Cohen et al. 2019 achieved accuracies of 93.1%, 82.3% and 88.6% in CN, MCI and AD, respectively [44]; however, their algorithm was not tested against unseen data. A review study concluded that, when compared to studies using cross-validation alone, studies using an unseen dataset for validation usually reported lower accuracy, especially when using a local population [68]. Recent studies have addressed the risk of overfitting for models built using a single dataset [71,72] and suggested conducting model validation using an independent dataset to report the model accuracy. In the current study, we reported accuracies for both validations: validation by an independent part of the same dataset (i.e., test 1) and by an unseen independent dataset (i.e., OASIS dataset). Our proposed FFNN with VRD features yielded 85.14%, 85.71% and 78.29% in CN, MCI and AD, respectively, in the Test 1 dataset, and 83.43%, 74.86% and 88% accuracies in CN, MCI and AD, respectively, in the OASIS test dataset. Both results were satisfactory and indicate the model’s capability to generalize to new data. 

### 4.5. Potential Clinical Application and Development of the Proposed Model

In the current study, the brain regional volumes and radiomics were retrieved from the MRI images manually using the chosen software. With the improved computer power and database management, script encoding is available. The retrieval of brain regional volumes and radiomics can be carried out after image acquisition in the image storage database. Together with the demographics obtained from the patient management system, the obtained features can be passed to the proposed neural network as the input for dementia classification. The predicted diagnosis from the neural network may help to triage AD and MCI patients from the CN and lead to a higher priority for clinicians to determine the diagnosis. 

### 4.6. Main Findings of Study

In this study, we utilized the brain regional volumes, radiomics retrieved from MPRAGE MRI images and patients’ demographics to build a classification for dementia patients; further, we evaluated the performance of the networks built in terms of overall accuracy, subclass accuracy, sensitivity and specificity. The proposed FFNN model using all three types of features demonstrated the best distinguishing ability and achieved very good performance in dementia classification. 

### 4.7. Study Limitations and Future Directions

In this study, two cohorts of neurodegenerative patients from a public database were used for model development and testing. The sample size was relatively small, even though it consisted of balanced samples in various groups. Small sample sizes provide less reliable estimates of the underlying data distribution, meaning that the developed model may miss subtle data patterns present in the data [70]. Further study is recommended using another independent local cohort of patients with a larger sample size to verify the proposed model. 

Radiomics of MPRAGE MRI images and demographic data were used as input to develop the classification model. In future studies, the model can be improved by incorporating image features from various imaging modalities, e.g., PET/CT with 18F-Flumetemetamol as a radionuclide for an amyloid study [73], T2-weighted MRI imaging for white matter hyper-intensity [74], arterial spin labeling MRI imaging for cerebral blood flow study [75] and resting state functional MRI imaging for interhemispheric functional connectivity [76], so as to develop a more comprehensive model. 

Furthermore, other clinical parameters, such as the Montreal Cognitive Assessment (MoCA) result, plasma amyloid-β level [77,78], can be included as input to develop or modify the networks, so as to improve the classification capabilities with more relevant parameters. 

## 5. Conclusions

This study established a feed-forward neural network model by integrating image features and demographics for various stages of dementia classification. The FFNN yielded good overall accuracies for MCI, AD and CN classification, with balanced subclass accuracy, sensitivity and specificity. The proposed FFNN model is simple and can be operated using a general-purpose computer in radiology departments. The application can be used as a reliable classification tool to prioritize patients with AD or MCI from CN. It may support the triage of patient for further testing, which shortens the diagnosis confirmation pathway.

## Figures and Tables

**Table 1:** Images collected from ADNI and OASIS databases.

	ADNI Dataset 21 Centers for Training	ADNI Dataset 4 Centers for Testing	OASIS Dataset for Testing
Alzheimer’s Disease	69	28	28
Mild Cognitive Decline	202	91	91
Health Control	135	57	57
Total	406	176	176

**Table 2:** Details for the 45 brain regional volumes.

45 Brain Regional Volumes Segmented by FreeSurfer		
Left-Lateral-Ventricle	Right-Lateral-Ventricle	CSF
Left-Inf-Lat-Vent	Right-Inf-Lat-Vent	Third-Ventricle
Left-Cerebellum-White-Matter	Right-Cerebellum-White-Matter	Forth-Ventricle
Left-Cerebellum-Cortex	Right-Cerebellum-Cortex	Fifth-Ventricle
Left-Thalamus	Right-Thalamus	Brain-Stem
Left-Caudate	Right-Caudate	WM-hypointensities
Left-Putamen	Right-Putamen	non-WM-hypointensities
Left-Pallidum	Right-Pallidum	Optic-Chiasm
Left-Hippocampus	Right-Hippocampus	CC_Posterior
Left-Amygdala	Right-Amygdala	CC_Mid_Posterior
Left-Accumbens-area	Right-Accumbens-area	CC_Central
Left-Ventral DC	Right-Ventral DC	CC_Mid_Anterior
Left-vessel	Right-vessel	CC_Anterior
Left-choroid-plexus	Right-choroid-plexus	
Left-WM-hypointensities	Right-WM-hypointensities	
Left-non-WM-hypointensities	Right-non-WM-hypointensities	

Inf: inferior; Lat: lateral; Mid: middle; DC: diencephalon; WM: white matter; CC: corpus callosum.

**Table 3:** Eight classes of radiomics features.

Radiomics Features	No. of Features
First-order statistics	14
2D-shaped based features	9
3D-shaped based features	13
Gray-level co-occurrence matrix (GLCM)	22
Gray-level run length matrix (GLRLM)	16
Gray-level size zone matrix (GLSZM)	16
Gray-level dependence matrix (GLDM)	12
Neighboring gray tone difference matrix (NGTDM)	5
**Total**	**107**

**Table 4:** Integration of patients’ demographics and image features.

	Radiomics 107 Features	Volumes 45 Features	Patients’ Demographics 2 Features	Total Number of Features
R only	✔			107
RD	✔		✔	109
V only		✔		45
VD		✔	✔	47
VRD	✔	✔	✔	154

**Table 5:** Demographics of the ADNI and OASIS datasets.

	ADNI 21 Centers for Training	ADNI 4 Centers for Testing	Oasis Dataset for Testing
Age range	55–90	65–90	74–89
Sex Ratio (M:F)	205:201	99:77	92:84
Alzheimer’s Disease	69	28	28
Mild Cognitive Decline	202	91	91
Health Control	135	57	57
Total	406	176	176

**Table 6:** Various features employed for model building using 4 algorithms. Training was the result when building the model using patients from ADNI 21 centers; Test 1 was the result when testing the model by patients from ADNI 4 centers; Oasis was the result when testing the model by patients from OASIS database. The red fonts highlight results over 70%. (**a**) Various features employed for model building using SVM. (**b**) Various features employed for model building using ensemble classifier (EC). (**c**) Various features employed for model building using decision tree (DT). (**d**) Various features employed for model building using feed-forward neural network (FFNN).

(**a**) Various features employed for model building using SVM.																	
**SVM**			**MCI**					**AD**					**CN**				
		**Overall** **Accuracy**	**Accuracy**	**Sensitivity**	**Specificity**	**Precision**	**F1 Score**	**Accuracy**	**Sensitivity**	**Specificity**	**Precision**	**F1 Score**	**Accuracy**	**Sensitivity**	**Specificity**	**Precision**	**F1 Score**
**R only**	Train	67.74%	73.45%	70.85%	76.67%	79.00%	74.70%	83.62%	52.63%	88.73%	43.48%	47.62%	78.41%	69.11%	82.50%	63.43%	66.15%
	Test1	40.57%	56.00%	56.99%	54.88%	58.89%	57.92%	74.29%	9.52%	83.12%	7.14%	8.16%	50.86%	26.23%	64.04%	28.07%	27.12%
	Oasis	35.43%	52.57%	58.54%	50.75%	26.67%	36.64%	66.29%	10.26%	82.35%	14.29%	11.94%	52.00%	35.79%	71.25%	59.65%	44.74%
**RD**	Train	62.03%	68.49%	63.88%	77.14%	84.00%	72.57%	81.14%	33.33%	83.77%	10.14%	15.56%	74.44%	63.03%	79.23%	55.97%	59.29%
	Test1	60.00%	66.86%	63.11%	75.47%	85.56%	72.64%	81.71%	0.00%	83.63%	0.00%	#DIV/0!	71.43%	57.14%	76.98%	49.12%	52.83%
	Oasis	54.29%	61.14%	57.05%	94.74%	98.89%	72.36%	83.43%	0.00%	83.91%	0.00%	#DIV/0!	64.00%	33.33%	67.52%	10.53%	16.00%
**V only**	Train	98.26%	98.51%	98.02%	99.00%	99.00%	98.51%	98.76%	97.06%	99.10%	95.65%	96.35%	99.26%	99.25%	99.26%	98.51%	98.88%
	Test1	70.29%	75.43%	75.82%	75.00%	76.67%	76.24%	84.57%	60.00%	85.29%	10.71%	18.18%	80.57%	64.56%	93.75%	89.47%	75.00%
	Oasis	61.14%	66.29%	68.24%	64.44%	64.44%	66.29%	76.57%	19.05%	84.42%	14.29%	16.33%	79.43%	65.22%	88.68%	78.95%	71.43%
**VD**	Train	94.79%	96.28%	93.02%	100.00%	100.00%	96.39%	96.03%	100.00%	95.43%	76.81%	86.89%	97.27%	95.56%	98.13%	96.27%	95.91%
	Test1	71.43%	74.86%	70.18%	83.61%	88.89%	78.43%	84.00%	#DIV/0!	84.00%	0.00%	#DIV/0!	84.00%	73.77%	89.47%	78.95%	76.27%
	Oasis	71.43%	71.43%	67.24%	79.66%	86.67%	75.73%	84.00%	#DIV/0!	84.00%	0.00%	#DIV/0!	87.43%	79.66%	91.38%	82.46%	81.03%
**VRD**	Train	81.14%	83.62%	78.15%	91.52%	93.00%	84.93%	89.58%	100.00%	88.83%	39.13%	56.25%	89.08%	82.61%	92.45%	85.07%	83.82%
	Test1	71.43%	73.14%	66.93%	89.58%	94.44%	78.34%	82.86%	0.00%	83.82%	0.00%	#DIV/0!	86.86%	86.96%	86.82%	70.18%	77.67%
	Oasis	68.00%	70.29%	67.27%	75.38%	82.22%	74.00%	80.00%	23.08%	84.57%	10.71%	14.63%	85.71%	80.77%	87.80%	73.68%	77.06%
(**b**) Various features employed for models building using ensemble classifier (EC).																	
**Ensemble**			**MCI**					**AD**					**CN**				
		**Overall** **Accuracy**	**Accuracy**	**Sensitivity**	**Specificity**	**Precision**	**F1 Score**	**Accuracy**	**Sensitivity**	**Specificity**	**Precision**	**F1 Score**	**Accuracy**	**Sensitivity**	**Specificity**	**Precision**	**F1 Score**
**R only**	Train	64.52%	68.98%	64.71%	76.35%	82.50%	72.53%	83.62%	54.84%	86.02%	24.64%	34.00%	76.43%	66.67%	80.42%	58.21%	62.15%
	Test1	49.14%	58.86%	57.14%	63.27%	80.00%	66.67%	78.29%	8.33%	83.44%	3.57%	5.00%	61.14%	35.14%	68.12%	22.81%	27.66%
	Oasis	58.29%	65.14%	62.39%	70.69%	81.11%	70.53%	81.14%	14.29%	83.93%	3.57%	5.71%	70.29%	54.90%	76.61%	49.12%	51.85%
**RD**	Train	67.49%	70.97%	66.27%	79.05%	84.50%	74.29%	83.62%	54.05%	86.61%	28.99%	37.74%	80.40%	74.77%	82.53%	61.94%	67.76%
	Test1	53.14%	61.71%	58.91%	69.57%	84.44%	69.41%	81.14%	22.22%	84.34%	7.14%	10.81%	63.43%	40.54%	69.57%	26.32%	31.91%
	Oasis	52.00%	60.57%	63.64%	58.16%	54.44%	58.68%	74.29%	20.69%	84.93%	21.43%	21.05%	69.14%	52.17%	80.19%	63.16%	57.14%
**V only**	Train	96.28%	96.28%	94.26%	98.45%	98.50%	96.33%	97.52%	96.83%	97.65%	88.41%	92.42%	98.76%	99.24%	98.53%	97.01%	98.11%
	Test1	73.14%	73.14%	69.03%	80.65%	86.67%	76.85%	82.86%	0.00%	83.82%	0.00%	#DIV/0!	90.29%	83.33%	93.91%	87.72%	85.47%
	Oasis	68.00%	69.71%	68.32%	71.62%	76.67%	72.25%	82.29%	36.36%	85.37%	14.29%	20.51%	84.00%	73.02%	90.18%	80.70%	76.67%
**VD**	Train	95.29%	96.28%	93.84%	98.96%	99.00%	96.35%	96.28%	100.00%	95.70%	78.26%	87.80%	98.01%	95.65%	99.25%	98.51%	97.06%
	Test1	77.91%	77.91%	73.21%	86.67%	91.11%	81.19%	83.72%	#DIV/0!	83.72%	0.00%	#DIV/0!	94.19%	86.67%	98.21%	96.30%	91.23%
	Oasis	70.86%	70.86%	67.57%	76.56%	83.33%	74.63%	84.57%	57.14%	85.71%	14.29%	22.86%	86.29%	78.95%	89.83%	78.95%	78.95%
**VRD**	Train	93.55%	94.29%	91.94%	96.88%	97.00%	94.40%	96.53%	100.00%	95.98%	79.71%	88.71%	96.28%	93.43%	97.74%	95.52%	94.46%
	Test1	74.86%	76.00%	70.00%	89.09%	93.33%	80.00%	84.00%	50.00%	84.39%	3.57%	6.67%	89.71%	86.79%	90.98%	80.70%	83.64%
	Oasis	69.71%	72.00%	74.12%	70.00%	70.00%	72.00%	80.57%	40.00%	88.97%	42.86%	41.38%	86.86%	78.33%	91.30%	82.46%	80.34%
(**c**) Various features employed for model building using decision tree (DT).																	
**Decision Tree**			**MCI**					**AD**					**CN**				
		**Overall** **Accuracy**	**Accuracy**	**Sensitivity**	**Specificity**	**Precision**	**F1 Score**	**Accuracy**	**Sensitivity**	**Specificity**	**Precision**	**F1 Score**	**Accuracy**	**Sensitivity**	**Specificity**	**Precision**	**F1 Score**
**R only**	Train	58.31%	65.51%	62.87%	69.28%	74.50%	68.19%	80.15%	36.59%	85.08%	21.74%	27.27%	70.97%	56.80%	77.34%	52.99%	54.83%
	Test1	47.43%	54.86%	54.55%	55.56%	73.33%	62.56%	76.00%	20.83%	84.77%	17.86%	19.23%	64.00%	40.00%	68.97%	21.05%	27.59%
	Oasis	54.86%	56.00%	53.99%	83.33%	97.78%	69.57%	85.14%	100.00%	84.97%	7.14%	13.33%	68.57%	60.00%	69.09%	10.53%	17.91%
**RD**	Train	62.78%	70.22%	65.50%	78.62%	84.50%	73.80%	82.63%	46.15%	83.85%	8.70%	14.63%	72.70%	59.09%	79.34%	58.21%	58.65%
	Test1	57.71%	64.00%	62.86%	65.71%	73.33%	67.69%	81.14%	22.22%	84.34%	7.14%	10.81%	70.29%	54.10%	78.95%	57.89%	55.93%
	Oasis	57.14%	61.14%	58.09%	71.79%	87.78%	69.91%	82.29%	36.36%	85.37%	14.29%	20.51%	70.86%	60.71%	72.79%	29.82%	40.00%
**V only**	Train	82.63%	87.34%	87.44%	87.25%	87.00%	87.22%	88.83%	65.00%	94.74%	75.36%	69.80%	89.08%	86.29%	90.32%	79.85%	82.95%
	Test1	62.86%	70.86%	68.93%	73.61%	78.89%	73.58%	78.29%	25.00%	85.16%	17.86%	20.83%	76.57%	65.38%	81.30%	59.65%	62.39%
	Oasis	64.57%	70.86%	70.10%	71.79%	75.56%	72.73%	78.29%	22.22%	84.71%	14.29%	17.39%	80.00%	68.33%	86.09%	71.93%	70.09%
**VD**	Train	84.12%	86.85%	86.57%	87.13%	87.00%	86.78%	88.34%	68.33%	91.84%	59.42%	63.57%	93.05%	87.32%	96.17%	92.54%	89.86%
	Test1	70.86%	74.86%	71.70%	79.71%	84.44%	77.55%	84.00%	50.00%	86.96%	25.00%	33.33%	82.86%	74.55%	86.67%	71.93%	73.21%
	Oasis	70.29%	74.29%	70.64%	80.30%	85.56%	77.39%	82.29%	41.18%	86.71%	25.00%	31.11%	84.00%	79.59%	85.71%	68.42%	73.58%
**VRD**	Train	71.22%	73.70%	68.65%	82.12%	86.50%	76.55%	84.86%	66.67%	86.02%	23.19%	34.41%	83.87%	77.17%	86.96%	73.13%	75.10%
	Test1	75.43%	75.43%	69.11%	90.38%	94.44%	79.81%	86.29%	83.33%	86.39%	17.86%	29.41%	89.14%	91.30%	88.37%	73.68%	81.55%
	Oasis	72.00%	75.43%	73.27%	78.38%	82.22%	77.49%	85.14%	55.00%	89.03%	39.29%	45.83%	83.43%	75.93%	86.78%	71.93%	73.87%
(**d**) Various features employed for model building using feed-forward neural network (FFNN).																	
**Feed Forward Neural Network**			**MCI**					**AD**					**CN**				
		**Overall** **Accuracy**	**Accuracy**	**Sensitivity**	**Specificity**	**Precision**	**F1 Score**	**Accuracy**	**Sensitivity**	**Specificity**	**Precision**	**F1 Score**	**Accuracy**	**Sensitivity**	**Specificity**	**Precision**	**F1 Score**
**R only**	Train	73.55%	75.76%	70.00%	85.71%	89.44%	78.54%	85.95%	78.95%	86.34%	24.19%	37.04%	85.40%	79.82%	87.95%	75.21%	77.45%
	Test1	51.43%	58.29%	57.26%	60.34%	74.44%	64.73%	82.86%	0.00%	83.82%	0.00%	#DIV/0!	61.71%	41.07%	71.43%	40.35%	40.71%
	Oasis	45.71%	54.86%	57.14%	53.06%	48.89%	52.69%	82.29%	28.57%	84.52%	7.14%	11.43%	54.29%	37.36%	72.62%	59.65%	45.95%
**RD**	Train	92.01%	92.29%	93.33%	91.26%	91.30%	92.31%	96.14%	87.10%	98.01%	90.00%	88.52%	95.59%	92.56%	97.11%	94.12%	93.33%
	Test1	55.43%	64.57%	62.96%	67.16%	75.56%	68.69%	76.57%	21.74%	84.87%	17.86%	19.61%	69.71%	54.55%	74.81%	42.11%	47.52%
	Oasis	55.43%	68.57%	73.97%	64.71%	60.00%	66.26%	74.86%	16.67%	84.11%	14.29%	15.38%	67.43%	50.00%	81.44%	68.42%	57.78%
**V only**	Train	100.00%	100.00%	100.00%	100.00%	100.00%	100.00%	100.00%	100.00%	100.00%	100.00%	100.00%	100.00%	100.00%	100.00%	100.00%	100.00%
	Test1	61.14%	64.57%	65.56%	63.53%	65.56%	65.56%	79.43%	16.67%	84.05%	7.14%	10.00%	78.29%	63.01%	89.22%	80.70%	70.77%
	Oasis	48.57%	49.71%	51.19%	48.35%	47.78%	49.43%	79.43%	27.78%	85.35%	17.86%	21.74%	68.00%	50.68%	80.39%	64.91%	56.92%
**VD**	Train	99.72%	99.72%	99.44%	100.00%	100.00%	99.72%	99.72%	100.00%	99.67%	98.41%	99.20%	100.00%	100.00%	100.00%	100.00%	100.00%
	Test1	65.71%	67.43%	67.74%	67.07%	70.00%	68.85%	77.14%	22.73%	84.97%	17.86%	20.00%	86.86%	78.33%	91.30%	82.46%	80.34%
	Oasis	54.29%	54.86%	55.24%	54.29%	64.44%	59.49%	77.71%	13.33%	83.75%	7.14%	9.30%	76.00%	63.64%	81.67%	61.40%	62.50%
**VRD**	Train	99.72%	99.72%	99.44%	100.00%	100.00%	99.72%	100.00%	100.00%	100.00%	100.00%	100.00%	99.72%	100.00%	99.57%	99.18%	99.59%
	Test1	76.57%	78.29%	76.53%	80.52%	83.33%	79.79%	89.71%	72.73%	92.16%	57.14%	64.00%	85.14%	78.18%	88.33%	75.44%	76.79%
	Oasis	73.14%	74.86%	78.75%	71.58%	70.00%	74.12%	88.00%	62.07%	93.15%	64.29%	63.16%	83.43%	71.21%	90.83%	82.46%	76.42%

**Table 7:** Five groups of features employed for model building using 4 algorithms. Training was the result when building the model using patients from ADNI 21 centers; Test 1 was the result when testing the model by patients from ADNI 4 centers; Oasis was the result when testing the model by patients from OASIS database. The red fonts highlighted the result over 70%. (**a**) Model performance using radiomics only in various model-building algorithms. (**b**) Model performance using RD in various model-building algorithms. (**c**) Model performance using volumes only in various model-building algorithms. (**d**) Model performance using VD in various model-building algorithms. (**e**) Model performance using VRD in various model-building algorithms.

(a) Model performance using radiomics only in various model-building algorithms																	
**R Only**			**MCI**					**AD**					**CN**				
		**Overall** **Accuracy**	**Accuracy**	**Sensitivity**	**Specificity**	**Precision**	**F1 Score**	**Accuracy**	**Sensitivity**	**Specificity**	**Precision**	**F1 Score**	**Accuracy**	**Sensitivity**	**Specificity**	**Precision**	**F1 Score**
**SVM**	Train	67.74%	73.45%	70.85%	76.67%	79.00%	74.70%	83.62%	52.63%	88.73%	43.48%	47.62%	78.41%	69.11%	82.50%	63.43%	66.15%
	Test1	40.57%	56.00%	56.99%	54.88%	58.89%	57.92%	74.29%	9.52%	83.12%	7.14%	8.16%	50.86%	26.23%	64.04%	28.07%	27.12%
	Oasis	35.43%	52.57%	58.54%	50.75%	26.67%	36.64%	66.29%	10.26%	82.35%	14.29%	11.94%	52.00%	35.79%	71.25%	59.65%	44.74%
**EC**	Train	64.52%	68.98%	64.71%	76.35%	82.50%	72.53%	83.62%	54.84%	86.02%	24.64%	34.00%	76.43%	66.67%	80.42%	58.21%	62.15%
	Test1	49.14%	58.86%	57.14%	63.27%	80.00%	66.67%	78.29%	8.33%	83.44%	3.57%	5.00%	61.14%	35.14%	68.12%	22.81%	27.66%
	Oasis	58.29%	65.14%	62.39%	70.69%	81.11%	70.53%	81.14%	14.29%	83.93%	3.57%	5.71%	70.29%	54.90%	76.61%	49.12%	51.85%
**DT**	Train	58.31%	65.51%	62.87%	69.28%	74.50%	68.19%	80.15%	36.59%	85.08%	21.74%	27.27%	70.97%	56.80%	77.34%	52.99%	54.83%
	Test1	47.43%	54.86%	54.55%	55.56%	73.33%	62.56%	76.00%	20.83%	84.77%	17.86%	19.23%	64.00%	40.00%	68.97%	21.05%	27.59%
	Oasis	54.86%	56.00%	53.99%	83.33%	97.78%	69.57%	85.14%	100.00%	84.97%	7.14%	13.33%	68.57%	60.00%	69.09%	10.53%	17.91%
**FFNN**	Train	73.55%	75.76%	70.00%	85.71%	89.44%	78.54%	85.95%	78.95%	86.34%	24.19%	37.04%	85.40%	79.82%	87.95%	75.21%	77.45%
	Test1	51.43%	58.29%	57.26%	60.34%	74.44%	64.73%	82.86%	0.00%	83.82%	0.00%	#DIV/0!	61.71%	41.07%	71.43%	40.35%	40.71%
	Oasis	45.71%	54.86%	57.14%	53.06%	48.89%	52.69%	82.29%	28.57%	84.52%	7.14%	11.43%	54.29%	37.36%	72.62%	59.65%	45.95%
(**b**) Model performance using RD in various model-building algorithms																	
**RD**			**MCI**					**AD**					**CN**				
		**Overall** **Accuracy**	**Accuracy**	**Sensitivity**	**Specificity**	**Precision**	**F1 Score**	**Accuracy**	**Sensitivity**	**Specificity**	**Precision**	**F1 Score**	**Accuracy**	**Sensitivity**	**Specificity**	**Precision**	**F1 Score**
**SVM**	Train	62.03%	68.49%	63.88%	77.14%	84.00%	72.57%	81.14%	33.33%	83.77%	10.14%	15.56%	74.44%	63.03%	79.23%	55.97%	59.29%
	Test1	60.00%	66.86%	63.11%	75.47%	85.56%	72.64%	81.71%	0.00%	83.63%	0.00%	#DIV/0!	71.43%	57.14%	76.98%	49.12%	52.83%
	Oasis	54.29%	61.14%	57.05%	94.74%	98.89%	72.36%	83.43%	0.00%	83.91%	0.00%	#DIV/0!	64.00%	33.33%	67.52%	10.53%	16.00%
**EC**	Train	67.49%	70.97%	66.27%	79.05%	84.50%	74.29%	83.62%	54.05%	86.61%	28.99%	37.74%	80.40%	74.77%	82.53%	61.94%	67.76%
	Test1	53.14%	61.71%	58.91%	69.57%	84.44%	69.41%	81.14%	22.22%	84.34%	7.14%	10.81%	63.43%	40.54%	69.57%	26.32%	31.91%
	Oasis	52.00%	60.57%	63.64%	58.16%	54.44%	58.68%	74.29%	20.69%	84.93%	21.43%	21.05%	69.14%	52.17%	80.19%	63.16%	57.14%
**DT**	Train	62.78%	70.22%	65.50%	78.62%	84.50%	73.80%	82.63%	46.15%	83.85%	8.70%	14.63%	72.70%	59.09%	79.34%	58.21%	58.65%
	Test1	57.71%	64.00%	62.86%	65.71%	73.33%	67.69%	81.14%	22.22%	84.34%	7.14%	10.81%	70.29%	54.10%	78.95%	57.89%	55.93%
	Oasis	57.14%	61.14%	58.09%	71.79%	87.78%	69.91%	82.29%	36.36%	85.37%	14.29%	20.51%	70.86%	60.71%	72.79%	29.82%	40.00%
**FFNN**	Train	92.01%	92.29%	93.33%	91.26%	91.30%	92.31%	96.14%	87.10%	98.01%	90.00%	88.52%	95.59%	92.56%	97.11%	94.12%	93.33%
	Test1	55.43%	64.57%	62.96%	67.16%	75.56%	68.69%	76.57%	21.74%	84.87%	17.86%	19.61%	69.71%	54.55%	74.81%	42.11%	47.52%
	Oasis	55.43%	68.57%	73.97%	64.71%	60.00%	66.26%	74.86%	16.67%	84.11%	14.29%	15.38%	67.43%	50.00%	81.44%	68.42%	57.78%
(**c**) Model performance using volumes only in various model-building algorithms																	
**V only**			**MCI**					**AD**					**CN**				
		**Overall** **Accuracy**	**Accuracy**	**Sensitivity**	**Specificity**	**Precision**	**F1 Score**	**Accuracy**	**Sensitivity**	**Specificity**	**Precision**	**F1 Score**	**Accuracy**	**Sensitivity**	**Specificity**	**Precision**	**F1 Score**
**SVM**	Train	98.26%	98.51%	98.02%	99.00%	99.00%	98.51%	98.76%	97.06%	99.10%	95.65%	96.35%	99.26%	99.25%	99.26%	98.51%	98.88%
	Test1	70.29%	75.43%	75.82%	75.00%	76.67%	76.24%	84.57%	60.00%	85.29%	10.71%	18.18%	80.57%	64.56%	93.75%	89.47%	75.00%
	Oasis	61.14%	66.29%	68.24%	64.44%	64.44%	66.29%	76.57%	19.05%	84.42%	14.29%	16.33%	79.43%	65.22%	88.68%	78.95%	71.43%
**EC**	Train	96.28%	96.28%	94.26%	98.45%	98.50%	96.33%	97.52%	96.83%	97.65%	88.41%	92.42%	98.76%	99.24%	98.53%	97.01%	98.11%
	Test1	73.14%	73.14%	69.03%	80.65%	86.67%	76.85%	82.86%	0.00%	83.82%	0.00%	#DIV/0!	90.29%	83.33%	93.91%	87.72%	85.47%
	Oasis	68.00%	69.71%	68.32%	71.62%	76.67%	72.25%	82.29%	36.36%	85.37%	14.29%	20.51%	84.00%	73.02%	90.18%	80.70%	76.67%
**DT**	Train	82.63%	87.34%	87.44%	87.25%	87.00%	87.22%	88.83%	65.00%	94.74%	75.36%	69.80%	89.08%	86.29%	90.32%	79.85%	82.95%
	Test1	62.86%	70.86%	68.93%	73.61%	78.89%	73.58%	78.29%	25.00%	85.16%	17.86%	20.83%	76.57%	65.38%	81.30%	59.65%	62.39%
	Oasis	64.57%	70.86%	70.10%	71.79%	75.56%	72.73%	78.29%	22.22%	84.71%	14.29%	17.39%	80.00%	68.33%	86.09%	71.93%	70.09%
**FFNN**	Train	100.00%	100.00%	100.00%	100.00%	100.00%	100.00%	100.00%	100.00%	100.00%	100.00%	100.00%	100.00%	100.00%	100.00%	100.00%	100.00%
	Test1	61.14%	64.57%	65.56%	63.53%	65.56%	65.56%	79.43%	16.67%	84.05%	7.14%	10.00%	78.29%	63.01%	89.22%	80.70%	70.77%
	Oasis	48.57%	49.71%	51.19%	48.35%	47.78%	49.43%	79.43%	27.78%	85.35%	17.86%	21.74%	68.00%	50.68%	80.39%	64.91%	56.92%
(**d**) Model performance using VD in various model-building algorithms																	
**VD**			**MCI**					**AD**					**CN**				
		**Overall** **Accuracy**	**Accuracy**	**Sensitivity**	**Specificity**	**Precision**	**F1 Score**	**Accuracy**	**Sensitivity**	**Specificity**	**Precision**	**F1 Score**	**Accuracy**	**Sensitivity**	**Specificity**	**Precision**	**F1 Score**
**SVM**	Train	94.79%	96.28%	93.02%	100.00%	100.00%	96.39%	96.03%	100.00%	95.43%	76.81%	86.89%	97.27%	95.56%	98.13%	96.27%	95.91%
	Test1	71.43%	74.86%	70.18%	83.61%	88.89%	78.43%	84.00%	#DIV/0!	84.00%	0.00%	#DIV/0!	84.00%	73.77%	89.47%	78.95%	76.27%
	Oasis	71.43%	71.43%	67.24%	79.66%	86.67%	75.73%	84.00%	#DIV/0!	84.00%	0.00%	#DIV/0!	87.43%	79.66%	91.38%	82.46%	81.03%
**EC**	Train	95.29%	96.28%	93.84%	98.96%	99.00%	96.35%	96.28%	100.00%	95.70%	78.26%	87.80%	98.01%	95.65%	99.25%	98.51%	97.06%
	Test1	77.91%	77.91%	73.21%	86.67%	91.11%	81.19%	83.72%	#DIV/0!	83.72%	0.00%	#DIV/0!	94.19%	86.67%	98.21%	96.30%	91.23%
	Oasis	70.86%	70.86%	67.57%	76.56%	83.33%	74.63%	84.57%	57.14%	85.71%	14.29%	22.86%	86.29%	78.95%	89.83%	78.95%	78.95%
**DT**	Train	84.12%	86.85%	86.57%	87.13%	87.00%	86.78%	88.34%	68.33%	91.84%	59.42%	63.57%	93.05%	87.32%	96.17%	92.54%	89.86%
	Test1	70.86%	74.86%	71.70%	79.71%	84.44%	77.55%	84.00%	50.00%	86.96%	25.00%	33.33%	82.86%	74.55%	86.67%	71.93%	73.21%
	Oasis	70.29%	74.29%	70.64%	80.30%	85.56%	77.39%	82.29%	41.18%	86.71%	25.00%	31.11%	84.00%	79.59%	85.71%	68.42%	73.58%
**FFNN**	Train	99.72%	99.72%	99.44%	100.00%	100.00%	99.72%	99.72%	100.00%	99.67%	98.41%	99.20%	100.00%	100.00%	100.00%	100.00%	100.00%
	Test1	65.71%	67.43%	67.74%	67.07%	70.00%	68.85%	77.14%	22.73%	84.97%	17.86%	20.00%	86.86%	78.33%	91.30%	82.46%	80.34%
	Oasis	54.29%	54.86%	55.24%	54.29%	64.44%	59.49%	77.71%	13.33%	83.75%	7.14%	9.30%	76.00%	63.64%	81.67%	61.40%	62.50%
(**e**) Model performance using VRD in various model-building algorithms																	
**VRD**			**MCI**					**AD**					**CN**				
		**Overall** **Accuracy**	**Accuracy**	**Sensitivity**	**Specificity**	**Precision**	**F1 Score**	**Accuracy**	**Sensitivity**	**Specificity**	**Precision**	**F1 Score**	**Accuracy**	**Sensitivity**	**Specificity**	**Precision**	**F1 Score**
**SVM**	Train	81.14%	83.62%	78.15%	91.52%	93.00%	84.93%	89.58%	100.00%	88.83%	39.13%	56.25%	89.08%	82.61%	92.45%	85.07%	83.82%
	Test1	71.43%	73.14%	66.93%	89.58%	94.44%	78.34%	82.86%	0.00%	83.82%	0.00%	#DIV/0!	86.86%	86.96%	86.82%	70.18%	77.67%
	Oasis	68.00%	70.29%	67.27%	75.38%	82.22%	74.00%	80.00%	23.08%	84.57%	10.71%	14.63%	85.71%	80.77%	87.80%	73.68%	77.06%
**EC**	Train	93.55%	94.29%	91.94%	96.88%	97.00%	94.40%	96.53%	100.00%	95.98%	79.71%	88.71%	96.28%	93.43%	97.74%	95.52%	94.46%
	Test1	74.86%	76.00%	70.00%	89.09%	93.33%	80.00%	84.00%	50.00%	84.39%	3.57%	6.67%	89.71%	86.79%	90.98%	80.70%	83.64%
	Oasis	69.71%	72.00%	74.12%	70.00%	70.00%	72.00%	80.57%	40.00%	88.97%	42.86%	41.38%	86.86%	78.33%	91.30%	82.46%	80.34%
**DT**	Train	71.22%	73.70%	68.65%	82.12%	86.50%	76.55%	84.86%	66.67%	86.02%	23.19%	34.41%	83.87%	77.17%	86.96%	73.13%	75.10%
	Test1	75.43%	75.43%	69.11%	90.38%	94.44%	79.81%	86.29%	83.33%	86.39%	17.86%	29.41%	89.14%	91.30%	88.37%	73.68%	81.55%
	Oasis	72.00%	75.43%	73.27%	78.38%	82.22%	77.49%	85.14%	55.00%	89.03%	39.29%	45.83%	83.43%	75.93%	86.78%	71.93%	73.87%
**FFNN**	Train	99.72%	99.72%	99.44%	100.00%	100.00%	99.72%	100.00%	100.00%	100.00%	100.00%	100.00%	99.72%	100.00%	99.57%	99.18%	99.59%
	Test1	76.57%	78.29%	76.53%	80.52%	83.33%	79.79%	89.71%	72.73%	92.16%	57.14%	64.00%	85.14%	78.18%	88.33%	75.44%	76.79%
	Oasis	73.14%	74.86%	78.75%	71.58%	70.00%	74.12%	88.00%	62.07%	93.15%	64.29%	63.16%	83.43%	71.21%	90.83%	82.46%	76.42%

## Data Availability

Publicly available datasets were analyzed in this study. The ADNI dataset can be found at: http://adni.loni.usc.edu/ (accessed on 22 January 2023). Data used in the preparation of this article were obtained from the Alzheimer’s Disease Neuroimaging Initiative (ADNI) database (adni.loni.usc.edu). As such, the investigators within the ADNI contributed to the design and implementation of ADNI and/or provided data but did not participate in the analysis or writing of this report. A complete listing of ADNI investigators can be found at: https://adni.loni.usc.edu/data-samples/access-data/#access_data (accessed on 22 January 2022). OASIS dataset: https://www.oasis-brains.org/#data. Approval was obtained on 28 February 2022 from the ADNI Data and Publications Committee (DPC) for the use of ADNI data for publication (accessed on 22 January 2023).
